# PReS-FINAL-2350: Overexpression of crem alpha leads to a higher inflammatory response in lps induced acute lung injury (ali) and might therefore trigger infectious complications in patients with autoimmune diseases

**DOI:** 10.1186/1546-0096-11-S2-P340

**Published:** 2013-12-05

**Authors:** E Verjans, A Wiener, K Ohl, N Wagner, S Uhlig, C Martin, K Tenbrock

**Affiliations:** 1 Pediatrics, RWTH Aachen University, Universitätsklinikum, Germany; 2 Institute of Pharmacology and Toxicology, RWTH Aachen University, Aachen, Germany

## Introduction

Patients with autoimmune diseases are highly susceptible towards infectious complications. In patients with SLE, infections are even one of the most common causes of morbidity, hospitalization and death. CREMα is a transcription factor, which is overexpressed in T cells from patients with systemic lupus erythematosus (SLE). Beyond this, CREMα is also upregulated in a murine model of LPS-induced acute lung injury (ALI).

## Objectives

It was our aim to examine whether the overexpression of CREMα leads to a higher inflammatory environment in a murine model of LPS-induced ALI and thus may contribute to infectious complications in patients with autoimmune diseases including SLE patients.

## Methods

ALI was induced via intratracheal LPS instillation in wild type and CREMα transgenic mice as well as in lymphopenic RAG-/- mice reconsituted with CREM-/- T cells. Lung functions and bronchial hyperresponsiveness (AHR) were measured with the flexiVent setup. The inflammatory phenotype was characterized by cell type analysis (FACS), cytokine expression (ELISA, qRT-PCR) and histology.

## Results

CREMα transgenic mice, which are characterized by a T cell-specific overexpression of CREMα, suffer from an enhanced development of LPS-induced ALI. CREMα overexpression thereby increases the number of T cells in bronchoalveolar lavage (BAL) and deteriorates lung function during the early phase of ALI. Furthermore CREMα transgenic mice show a stronger inflammatory response with higher levels of TNFα, IL-6 and IL-17 correlating with increased numbers of T cells and neutrophils in BAL. Vice versa, expression of FoxP3 and IL-2 and the numbers of regulatory T cells are downregulated in lung tissue as well as in the BAL. These changes result in restricted lung function and thereby reduced oxygenation of the animals. Beside this, an adoptive transfer of CREM-/- CD4+ T cells resulted in ameliorated disease levels in RAG -/- compared to RAG-/- mice transferred with wild type CD4+ T cells.

## Conclusion

Thus, CREMα-transgenic animals represent a model in which proinflammatory T cells aggravate ALI. Given the fact that patients with autoimmune diseases like SLE show higher levels of CREMα and an increased susceptibility towards infectious complications our finding is potentially of clinical significance and enables new therapeutical strategies.

## Disclosure of interest

None declared.

